# Supplementary material for: WordCommentsAnalyzer: A windows software tool for qualitative research
Source: F1000Res. 2018 Sep 4;7:536. Originally published 2018 May 3. [Version 2] doi: 10.12688/f1000research.14819.2 (PMC6137411; doi:10.12688/f1000research.14819.2)
Supplement: Supplementary file 3 [file f1000research-7-17614-s0002.tgz › b2a79496-4ada-47c4-9682-a99ce867836d.zip!/Eight Iranian Health Journals/Journal of Knowledge and Health_4.docx]

© Journal of Knowledge and Health. This work is licensed under CC BY 4.0 (https://creativecommons.org/licenses/by/4.0/).

Volume & Issue: دوره 12، شماره 2: 1396

Title: رده‌بندي نتایج آنژیوگرافی با ترکیب شبکه عصبی- فازی و الگوریتم ژنتیک

http://knh.shmu.ac.ir/index.php/site/article/view/1669

مقدمه: بیماری عروق کرونری قلب ازجمله بیماری‌هایی است که تشخیص زودهنگام آن امری حیاتی است. مطالعات نشان می‌دهد روش‌های نوین هوش مصنوعی در پیش‌بینی و تشخیص بیماری‌ها به‌طور گسترده موردتوجه محققان قرارگرفته است. هدف از این پژوهش، رده‌بندي نتایج آنژیوگرافی به دو دسته طبيعي و غیرطبيعي با استفاده از روش‌های هوش مصنوعی است. مواد و روش‌ها: در این مطالعه از یک مجموعه داده شامل 152 بیمار که تحت آنژیوگرافی عروق کرونری قلب قرارگرفته بودند، استفاده شد. به‌منظور رده‌بندي نتایج حاصل از آنژیوگرافی عروق کرونری قلب از ترکیب شبکه عصبی- فازی و الگوریتم ژنتیک استفاده شد. سیستم پیشنهادی در نرم‌افزار MATLAB پیاده‌سازی شد و مورد ارزیابی قرار گرفت. نتایج: در پیاده‌سازی انجام‌شده، از 85 درصد داده‌ها جهت مرحله آموزش شبکه عصبی- فازی و 15 درصد باقیمانده جهت مرحله آزمون استفاده شد. نتایج حاصل از شبیه‌سازی در شاخص‌های صحت، دقت، حساسیت و اختصاصیت در حالت میانگین به‌ترتیب 9496/0، 9253/0، 9435/0 و 9569/0 و در بهترین حالت برای هر شاخص معادل عدد 1 به‌دست‌آمده است. نتیجه‌گیری: استفاده از الگوریتم ژنتیک در فرآيند آموزش شبکه عصبی- فازی موجب بهبود سرعت در این شبیه‌سازی شد. همچنین بالا بودن شاخص‌های به‌دست‌آمده عملکرد مناسب سیستم پیشنهادی در رده‌بندي و تشخیص افراد مبتلا‌به بیماری عروق کرونری قلب را تأیید می‌کند.

Title: مقایسه تأثير تمرینات عمومی و اختصاصی کمری- لگنی بر کنترل حرکت و درد در افراد دارای کمردرد مزمن

http://knh.shmu.ac.ir/index.php/site/article/view/1683

مقدمه: هدف تحقیق حاضر مقایسه تأثير تمرینات عمومی و اختصاصی منطقه کمری- لگنی بر درد و کنترل حرکت کمری- لگنی در افراد دارای کمردرد بود. مواد و روش‌ها: جامعه آماری تحقیق شامل بیماران مبتلا‌به کمردرد مزمن غیراختصاصی بودند که 44آزمودنی با دامنه سنی 30 تا 40 سال برای انجام تحقیق فراخوانده شدند. باتوجه به اعمال مداخله، وجود گروه کنترل و انتخاب هدفمند آزمودنی‌ها به‌علت ماهیت تحقیق، روش تحقيق حاضر از نوع نيمه تجربي است. قبل و بعد از اجرای دو نوع متفاوت پروتکل تمرینی عمومی و اختصاصی کمری- لگنی، سنجش درد و کنترل حرکت به‌ترتیب با استفاده از مقیاس بصری درد و آزمون‌های لوماجوکی از آزمودنی‌ها به‌عمل آمد. نتایج با استفاده از تجزيه و تحليل واریانس يك‌راهه،تحليل كوواريانس، تی وابسته و مستقل در سطح معنی‌داری 05/0 در نرم‌افزار SPSS نسخه 22 مورد تحلیل قرار گرفت. نتايج: نتایج نشان داد که پروتکل تمرینات عمومی باعث کاهش کمردرد به ميزان 37% و بهبود كنترل حركت كمر به ميزان 39% گرديد درحالي‌كه اين ميزان در پروتكل اختصاصي به‌ترتيب 52% و 53% بود كه نشان مي‌دهد برتری در هر دو مورد با تمرینات اختصاصی مي‌‌باشد. نتیجه‌گیری: نتیجه این مطالعه نشان داد سامانه کنترل حرکتی در بیماران مبتلا‌به کمردرد از انعطاف‌پذیری کافی برخوردار نبوده و درنتیجه این افراد جهت حفظ ثبات حرکتی خود ناگزیر از اتخاذ استراتژی‌هایی هستند که خود مي‌‌توانند پیامدهای جدید مخربی برای این بیماران در پی داشته باشند. بعد از انجام تمرینات اختصاصی کمری- لگنی، تغییرات قابل توجهی در درد و کنترل حرکت کمر به‌وجود آمده است که مي‌‌تواند مورد توجه محققان آینده جهت کمک به مراقبت و پیشگیری در بیماران مبتلا‌به کمردرد قرار بگیرد.

Title: تأثير هشت هفته تمرین هوازی بر مقاومت به انسولین، ترکیب بدن و توان هوازی دختران دانشجوی دارای اضافه وزن

http://knh.shmu.ac.ir/index.php/site/article/view/1670

مقدمه: شیوع چاقی به‌عنوان یک مشکل بهداشتی در سراسر جهان شناخته شده است و به موازات آن، بیماري‌هاي مرتبط با چاقی، از جمله مقاومت به انسولین، سندرم متابولیک، دیابت نوع 2، فشار خون بالا و بیماري‌هاي قلبی عروقی نیز شیوع یافته است. در این مطالعه تأثير هشت هفته تمرین هوازی بر مقاومت به انسولین، ترکیب بدن و آمادگی قلبی تنفسی زنان دارای اضافه وزن بررسی شد. مواد و روش‌ها: در این مطالعه نیمه تجربی، 21 زن دارای اضافه وزن سالم با دامنه سنی 20 تا 30 سال به‌طور تصادفی در دو گروه تجربی (10 نفر) و کنترل (11 نفر) تقسیم شدند و به مدت هشت هفته به انجام تمرین هوازی پرداختند. برنامه تمرین هوازی شامل هشت هفته با تواتر پنج جلسه در هفته و هر جلسه به مدت 30 دقیقه با شدت 55 تا 75 درصد ضربان قلب ذخیره‌ای بود. گلوکز و انسولین ناشتا، HOMA-IR، شاخص‌های تن‌سنجی و حداکثر اکسیژن مصرفی قبل و بعد از هشت هفته اندازه‌گیری شد. نتایج: کاهش معنی‌دار وزن (048/0=P) و شاخص توده بدن (002/0=P)، از طرفی افزایش معنی‌دار حداکثر اکسیژن مصرفی (000/0=P) در گروه تجربی مشاهده شد. اما اين تغييرات در گروه کنترل معني‌دار نبود. تنها تفاوت ميانگين‌هاي وزن (003/0=P) و شاخص توده بدن (025/0=P) بين دو گروه معني‌داري بود. نتیجه‌گیری: تمرين هوازی در دختران داراي اضافه وزن تأثيرات مفيـدي بـر وزن و نمايه تـوده بـدن دارد. با این حال، این مداخلات می‌توانند به‌منظور کمک به پیشگیری از ابتلا به بیماری‌های مزمن در افراد چاق و دارای اضافه وزن مؤثر باشد.

Title: ارتباط بین ظرفیت تام آنتی‌اکسیدان با حجم‌های استاتیک معرف عملکرد ریوی در مردان بزرگسال مبتلا‌به آسم مزمن

http://knh.shmu.ac.ir/index.php/site/article/view/1652

مقدمه: شواهد اخیر نشان می‌دهند که بیماران مبتلا‌به آسم از ظرفیت آنتی‌اکسیدانی پایین تری نسبت به افراد سالم برخوردارند. با این وجود، اطلاعات محدودی در خصوص چگونگی تأثیر آن بر عملکرد ریوی در این بیماران وجود دارد. مطالعه حاضر با هدف تعیین ارتباط بین ظرفیت تام آنتی‌اکسیدان با شاخص‌های اسپیرومتری در اين بیماران انجام شد. مواد و روش‌ها: برای این منظور، 30 مرد بزرگسال غیرفعال مبتلا‌به آسم با شدت خفیف تا متوسط به شیوه هدفمند وارد مطالعه شدند و نمونه‌های خون ناشتا با هدف اندازه‌گیری ظرفیت تام آنتی‌اکسیدان از آنها اخذ شد. همچنين شاخص‌های آنتروپومتریکی، ظرفیت حیاتی با فشار (FVC)، حداکثر حجم بازدمی با فشار در ثانیه اول(FEV1) و نسبت FVC/FEV1 به‌عنوان مارکرهای اسپیرومتری معرف عملکرد ریوی نیز اندازه‌گیری شد. از تجزيه و تحليل همبستگی پیرسون جهت تعیین ارتباط بین ظرفیت تام آنتی‌اکسیدان با شاخص‌های اسپیرومتری استفاده گردید. نتایج: بر پایه یافته‌های آماری، ارتباط مستقیم و معنی‌داری بین ظرفیت تام آنتی‌اکسیدان با FVC (036/0=P، 48/0=r)، FEV1 (002/0=P، 73/0=r) و FVC/FEV1 (001/0=P، 77/0=r) مشاهده شد. نتیجه‌گیری: بر پایه این شواهد اینگونه نتیجه‌گیری می‌شود که ظرفیت تام آنتی‌اکسیدان یک شاخص پیشگوی مناسب برای تعیین عملکرد ریوی و شدت بیماری در مردان مبتلا‌به آسم است.

Title: بررسی مقایسه‌ای کارآیی جذب رنگ Methylene Blue با استفاده از گرانول مرجان آهکی و گرانول لیکا از فاضلاب مصنوعی

http://knh.shmu.ac.ir/index.php/site/article/view/1721

مقدمه: رنگ‌ها از جمله آلاینده‌های اصلی موجود در فاضلاب‌های صنایع نساجی مي‌باشند که به‌دلیل ساختار پیچیده مولکولی، غالباً سمی و سرطان‌زا بوده و در محیط زیست پایدار مي‌باشند. هدف از انجام این پژوهش مقایسه کارایی مرجان آهکی و لیکا در حذف رنگ متیلن بلو از پساب سنتتیک مي‌باشد. مواد و روش‌ها: این مطالعه یک مطالعه بنیادی-کاربردی مي‌باشد که در آن از مرجان آهکی و لیکا به‌عنوان جاذب‌های مؤثر در حذف رنگ و بار آلی و رنگ متیلن بلو استفاده شده است. شاخص‌های مورد بررسی شامل وزن جاذب، زمان تماس، غلظت رنگ‌زا و pH بود. همچنین داده‌های حاصل از ثابت تعادل با استفاده از مدل‌های ایزوترمی و سینتیکی مورد بررسی قرار گرفت. نتایج: نتایج آزمایشات نشان داد كه میزان جذب رنگ توسط هر دو نوع جاذب با افزایش pH، زمان تماس و میزان جاذب افزایش مي‌یابد. افزایش غلظت رنگ‌زا از 50 میلی‌گرم در لیتر به 100 و 150 میلی‌گرم در لیتر باعث کاهش راندمان حذف رنگ و بار آلی گردید. نتایج نشان داد که داده‌های ثابت تعادل از هر دو نوع مدل ایزوترمی تبعیت مي‌کنند، با این تفاوت که مدل ایزوترم لانگموئر ضریب همبستگی بالاتری در استفاده از جاذب مرجان آهکی (1=R2)، و مدل فرندلیخ ضریب همبستگی بالاتری (999/0=R2) در استفاده از جاذب لیکا نشان داد. همچنین بررسی داده‌ها نشان داد که سینتیک حذف رنگ متیلن بلو از هر دو نوع مدل سینتیکی تبعیت مي‌کند که در این مورد نیز معادله سینتیکی درجه دوم ضریب همبستگی بالاتری (870/0<R2) را از خود نشان داد. نتیجه‌گیری: باتوجه به نتایج به‌دست آمده مشخص گردید که گرانول مرجان آهکی در حذف رنگ متیلن بلو نسبت‌به گرانول لیکا کارآیی بهتری دارد. استفاده از این جاذب‌ها به علت ته‌نشینی سریع و عدم باقی مانده مؤثر در پساب مي‌تواند کاربرد آسان‌تری نسبت‌به سایر جاذب‌ها داشته باشد.

Title: بررسی ادغام ژنی AML1/ETO در کودکان مبتلا‌به لوسمی مراجعه‌کننده به بیمارستان امام خمینی (ره) تهران

http://knh.shmu.ac.ir/index.php/site/article/view/1677

مقدمه: ادغام (T(8;21 یا به عبارتی AML1-ETO شایع‌ترین ترانسلوکاسیون در لوسمی میلوئیدی حاد در هر دو سن کودکان و بزرگسالان است. وقوع این ادغام ژنی در آینده درمانی شخص بیمار تأثیرگذار است و نقش پیش‌آگهی دارد. در جامعه مبتلایان ایرانی به لوسمی حاد این ادغام ژنی مشخص نشده و شیوع آن نامشخص است. لذا مطالعه حاضر با هدف بررسی ادغام ژنی AML1/ETO در کودکان مبتلا‌به لوسمی مراجعه‌کننده به بیمارستان امام خمینی(ره) تهران انجام شد. مواد و روش‌ها: در این مطالعه بررسی مقطعی تعداد 58 کودک مبتلا‌به لوسمی میلوئیدی حاد با ریخت‌شناسی متفاوت در طی سال 95 مورد بررسی قرار گرفتند. پس از تهیه خون محیطی، بلافاصله سلول‌های تک‌هسته‌ای بیماران جدا شدند. همچنین لام خون محیطی افراد برای تأيید ابتلا به لوسمی تهیه شد. از سلول‌های تک‌هسته‌ای بیماران RNA تام بیماران استخراج شد. با استفاده از روش‌های مولکولی PCR, Nested PCR همین‌طور Real-time PCR جهت ارزیابی ادغام مورد استفاده قرار گرفت. نتایج: نتایج مطالعه حاضر نشان داد که رنج سنی کودکان مورد مطالعه 5-4 سال بود. نسبت جنسیتی 5/53% مؤنث و 5/46% مذکر بودند. 80% افراد مورد مطالعه دارای این ادغام ژنی بودند و همین‌طور mRNA آن در پلاسما در حد قابل توجهی وجود داشت. در بررسی خون محیطی غلظت هموگلوبین 6/9 گرم در دسی‌لیتر و میانگین شمارش گلبول‌های سفید 70500 در میکرولیتر به‌دست آمد. نتیجه‌گیری: با‌توجه به فراوانی ادغام این ترانسلوکاسیون ژنی در جمعیت ایرانی مورد مطالعه، به‌نظر می‌آید استفاده از بررسی این ادغام جهت تعیین پیش‌آگهی و نیز استراتژی درمانی مفید باشد.

Title: اثر فعالیت در آب و مقایسه آن با تمرین تراباند بر کاربردی‌ترین آزمون‌های عملکردی در مردان سالمند مبتلا‌به استئوآرتریت زانو

http://knh.shmu.ac.ir/index.php/site/article/view/1688

مقدمه: سالمندی مرحله‌ای از سیر طبیعی زندگی انسان است که استئوآرتریت در آن بیشتر به چشم می‌خورد. این مطالعه به‌منظور تعيين اثر فعالیت در آب و مقایسه آن با تمرین تراباند بر کاربردی‌ترین آزمون‌های عملکردی در مردان سالمند مبتلا‌به استئوآرتریت زانو انجام گرفت. مواد و روش‌ها: جامعه آماری، مردان سالمند مبتلا‌به استئوآرتریت زانو بود که به‌روش تصادفی ساده و از نمونه‌های در دسترس، تعداد 36 نفر به‌صورت تصادفی به سه گروه 12 نفری (تراباند، فعالیت در آب و کنترل) تقسیم شدند. آزمودنی‌ها در دو گروه تراباند و فعالیت در آب به مدت 6 هفته، 4 جلسه در هفته برنامه‌های تمرینی مربوط‌به گروه خود را اجرا نمودند. برای اندازه‌گیری آزمون‌های عملکردی از سه آزمون عملکردی استفاده گردید. برای بررسی نتایج از آزمون‌های تحلیل واریانس يك طرفه و آزمون تی‌ زوجي استفاده شد. داده‌های حاصل از پژوهش با استفاده از نرم‌افزار SPSS، در سطح معنی‌داری 5 درصد 05/0≤α مورد تحلیل قرار گرفت. نتايج: نتایج تحقیق حاضر درخصوص شاخص‌های عملکردی (بالارفتن از پله، 30 ثانیه نشست و برخاست و 8 فوت راه‌رفتن)، بین گروه‌های تراباند و فعالیت در آب با گروه کنترل اختلاف معنی‌دار بود (05/0=P). همچنین مقایسه درون‌گروهی در گروه تراباند و فعالیت در آب در متغیرهای اندازه‌گیری شده، اختلاف معنی‌داری را نشان داد (05/0=P). نتیجه‌گیری: نتایج این مطالعه نشان داد که شش هفته تمرین تراباند و فعالیت در آب می‌تواند سبب بهبود در آزمون‌های عملکردی مردان سالمند مبتلا‌به استئوآرتریت زانو شود.

Title: پیش‌بینی عوامل مرتبط با ابتلا به دیابت نوع دو با استفاده از رگرسیون لجستیک

http://knh.shmu.ac.ir/index.php/site/article/view/1689

مقدمه: دﯾﺎﺑﺖ ﯾﮏ ﺑﯿﻤﺎري ﺑﺎ ﺷﯿﻮع ﺑﺎﻻ است که ﻋﻮارض آن ﺑﻪ‌ﻃﻮر اوﻟﯿﻪ ﻣﻮﺟﺐ آﺳـﯿﺐ ﺑـﻪ ﺳﯿﺴـﺘﻢ ﻋﺮوﻗـﯽ ﻣﯽﺷﻮد. هدف از این مطالعه پیش‌بینی عوامل مرتبط با ابتلا به دیابت نوع دو با استفاده از رگرسیون لجستیک می‌باشد. مواد و روش‌ها: اين مطالعه از نوع مقطعي- تحلیلی بوده و و داده‌هاي آن مربوط‌به مطالعه مشهد مي‌باشد که از سال 1387 آغاز و تاکنون ادامه دارد. جمعیت مورد مطالعه کلیه افراد 35 تا 65 سال ساکن شهر مشهد است و نمونه‌ها نيز شامل 9386 نفر مي‌باشند كه با استفاده از روش نمونه‌گیری طبقه‌ای- خوشه‌ای انتخاب شدند. در اين مطالعه اطلاعات دموگرافیک، شاخص تن‌سنجی، فشارخون، اضطراب، افسردگی، سطح فعالیت فیزیکی، الگوهای غذایی و عوامل التهابی، خونی و بیوشیمی ثبت شدند. برای تحلیل داده‌ها از نرم‌افزار SPSS استفاده گردید. سطح معنی‌داری 05/0 در نظر گرفته شد. مدل رگرسیون لجستیک به‌منظور بررسی عوامل مرتبط با ابتلا به دیابت نوع دو بر داده‌ها برازش داده شد. نتایج: براساس نتايج مطالعه، شیوع دیابت نوع دو 4/14% (1387 نفر) بود. نتایج همچنين نشان داد كه بین سن، شاخص تن‌سنجی، فشارخون، افسردگی، الگوهای غذایی و ابتلا به دیابت نوع دو از لحاظ آماری اختلاف معنی‌داري وجود دارد (05/0P<). علاوه بر اينها، عوامل التهابی، خونی و بیوشیمی نيز در ابتلا به ديابت نوع دو تأثير معنی‌داري داشتند (05/0P<). نتيجه‌گيري: بخش عمده‌ای از عوامل مرتبط با ابتلا به دیابت نوع دو، متغیرهای قابل کنترل مربوط‌به سبک زندگی می‌باشند، بنابراین بهتر است اقدامات صورت گرفته در حوزه آموزش عمومی و پیشگیری در جهت ارتقای شیوه زندگی سالم در سطح جامعه متمرکز گردند.

Title: اثر یک دوره تمرینات هوازی با شدت متوسط بر فعالیت آمینوترانسفرازهای کبدی در زنان چاق بزرگسال کم تحرک

http://knh.shmu.ac.ir/index.php/site/article/view/1719

مقدمه: چاقی به‌عنوان عامل خطر کبد چرب و سندرم متابولیک شناخته شده است. این مطالعه با هدف تعیین اثر یک دوره تمرینات هوازی بر فعالیت آمینوترانسفرازهای کبدی (AST, ALT) و آلکالین فسفاتاز (ALP) در زنان چاق بزرگسال انجام گرفت. مواد و روش‌ها: در این مطالعه نیمه تجربی، 28 زن بزرگسال چاق کم تحرک (36≥BMI≥30) به شیوه تصادفی به دو گروه ورزش (14=n) و کنترل (14=n) تقسیم شدند. گروه ورزش در یک دوره تمرینات هوازی به مدت 12 هفته به تعداد 3 جلسه در هفته در دامنه شدت 60 تا 75 درصد ضربان قلب بیشینه شرکت نمودند. فعالیت AST، ALT و ALP همچنین شاخص‌های آنتروپومتریکی در شرایط قبل و پس از مداخله تمرینی اندازه‌گیری و توسط آزمون‌های آماری تی مستقل و همبسته با یکدیگر مقایسه شدند. سطح معنی‌داری 05/0 در نظر گرفته شد. نتایج: در شرایط پایه، تفاوت معنی‌داری در فعالیت آنزیم‌های کبدی و شاخص‌های آنتروپومتریکی بین دو گروه مشاهده نشد (05/0≤P). در مقایسه با پیش آزمون، علیرغم عدم‌تغییر فعالیت ALP (114/0=P)، کاهش معنی‌داری در فعالیت AST (023/0≥P) و ALT (011/0≥P) به‌واسطه تمرین هوازی در گروه ورزش مشاهده شد. تمرینات هوازی به کاهش معنی‌دار شاخص‌های آنتروپومتریکی در گروه ورزش منجر شد (05/0≥P). در گروه کنترل، هیچ تغییری در متغیرها بین دو شرایط پیش و پس آزمون مشاهده نشد (05/0≤P). نتیجه‌گیری: تمرین هوازی منظم با اثرات درمانی ایمن و مؤثر روی فعالیت آنزیم‌های کبدی در زنان چاق همراه است. این فواید را می‌توان به کاهش توده چربی به‌ویژه چربی شکمی در پاسخ مداخله تمرینی در افراد چاق نسبت داد.
